# The Structure of Bitumen: Conceptual Models and Experimental Evidences

**DOI:** 10.3390/ma15030905

**Published:** 2022-01-25

**Authors:** Michele Porto, Ruggero Angelico, Paolino Caputo, Abraham A. Abe, Bagdat Teltayev, Cesare Oliviero Rossi

**Affiliations:** 1 Department of Chemistry and Chemical Technologies, University of Calabria, Via P. Bucci, Cubo 14/D, 87036 Arcavacata di Rende, CS, Italy; michele.porto@unical.it (M.P.); abraham.abe@unical.it (A.A.A.); cesare.oliviero@unical.it (C.O.R.); 2 Department of Agricultural, Environmental and Food Sciences (DIAAA), University of Molise, Via de Sanctis, 86100 Campobasso, CB, Italy; 3 JSC “Kazakhstan Highway Research Institute”, Almaty 050061, Kazakhstan; bagdatbt@yahoo.com

**Keywords:** crude oil, asphalt binder, bitumen, asphaltenes, colloids, fractals, scattering techniques

## Abstract

Bitumen, one of the by-products of petroleum industry processes, is the most common binder used in road pavements and in the construction industry in general. It is a complex organic mixture of a broad range of hydrocarbons classified into four chemical families, collectively known with the acronym SARA fractions, which include saturates, aromatics, resins and asphaltenes. Since the 1940s, researchers working on bitumen and the science behind its existence, nature and application have investigated the spatial organization and arrangement of several molecular species present in the binder. Therefore, several models have been proposed in the literature, and they are more or less corroborated by experimental studies, although most of them are model-dependent; for example, the structural investigations based on scattering techniques. One of the most popular models that has met with a wide consensus (both experimentally and of the modeling/computational type) is the one aiming at the colloidal description of bitumen’s microstructure. Other types of models have appeared in the literature that propose alternative views to the colloidal scheme, equally valid and capable of providing results that comply with experimental and theoretical evidence. Spurred by the constant advancement of research in the field of bitumen science, this literature review is aimed at providing a thorough, continuous and adept state of knowledge on the modeling efforts herein elaborated, in order to more precisely describe the intricacy of the bituminous microstructure. In this body of work, experimental evidence, along with details of bitumen’s microstructure (depicting the colloidal state of bitumen), is particularly emphasized. We will also try to shed light on the evolution of the experimental and theoretical results that have focused on the aspect of the association and aggregation properties of asphaltenes in various models and real systems.

## 1. Introduction

Asphalt binder is the gluey material that keeps the mineral aggregate particles together in the composite used in road paving processes. It is the product of a refinery operation whereby a residuum obtained after the distillation of crude oil is treated by air blowing or by a solvent method to produce a product that meets specifications for a variety of road/highway construction and other uses [1,2]. Bitumen is also the name given in Europe to a manufactured product, while asphalt binder is used in North America and many other countries.

Nowadays, a significant amount of bitumen derived from petroleum is channeled into road paving processes, while the rest is utilized for other construction processes, such as painting, varnishes, roofing, insulation, the production of rust-protective compositions, battery boxes, and as one of the starting materials that go into the production of rubber products, brake linings and fuel briquettes. A variety of standard tests are available to define the quality and viscosity specifications of bitumen [2]. Asphalt binder can be fractionated into four general (but not chemical) classes of compounds (SARA) [3]: (i) saturate hydrocarbons, correlated with the softening point of the material and empirically measured by the penetration index; (ii) aromatic and naphthene constituents, which are partially hydrogenated aromatic compounds; (iii) resin constituents, in some cases referred to as polar aromatic constituents, which contain a variety of alkyl residues and functional groups, and (iv) asphaltene molecules, constituted by a conjugated carbon core, having functional groups and heterocyclic compounds and carrying alkyl sidechains grafted directly to these cores. Asphaltenes (henceforth the term asphaltene(s) will be denominated with the acronym ASP) are associated with highly aromatic (H/C~1.0–1.3) and high-molecular weight (MW) molecules [4], while resins have a higher H/C ratio (1.2–1.7), lower aromaticity, and lower MW compared to ASPs [5,6].

Among the other fractions of bitumen, ASPs are the least soluble fraction of an almost continuous spectrum of several polyaromatic molecular species and can be extracted or separated from the other fractions through precipitation via the addition of a large volume of n-alkane to a crude oil (ASTM protocol D6560) [7].

The term resin is generally used to refer to a substance that has undergone elution from several solid adsorbents, while the term maltenes (petrolenes or de-asphalted oil) refers to a mixture of the resin and oil constituents that are obtained from the filtrates via the precipitation of ASPs [8].

ASPs are often referred to as the “cholesterol of petroleum” [9], a term borrowed from medical terminology to define the clogging effect occurring in wellbores and transportation pipelines caused by the precipitation, deposition and dense flocculation of ASPs subjected to certain physicochemical conditions [10]. Each year, the damage caused by the deposition of ASPs results in the loss of billions of dollars in the oil industry, which also is reflected in reduced production capacity, wells prematurely shutting-in, and implications for management techniques [11]. ASPs are therefore the lyophobic components of bitumen, and their dispersion is affected by several factors such as the nature of the dispersing medium (paraffinic or aromatic), as well as the chemical structure and relative proportion of resins and ASPs [5,12,13,14]. Indeed, a high content of resins imparts to a product an adhesive property and plasticity, whereas high ASP content is usually responsible for harder, more brittle bitumen, as evidenced from the structure and rheological properties of modified bitumen. Bitumen containing a high resin content translates to higher product adhesiveness, as well as a high level of plasticity, while on the other hand, a high ASP content imparts hardness and results in a more brittle bitumen [15,16,17,18,19]. Furthermore, the presence of ASPs is usually responsible for a significant increment in the viscosity of crude oil [20,21], thus making its transportation and processing more difficult. These properties and many others are dictated by the microstructure of bitumen, that is, the structure and dynamics of ASP aggregates dispersed in the maltene moiety.

The purpose of this review is to comprehensively illustrate the evolution of models and experimental progresses in the studies of ASP aggregation mechanisms by emphasizing its colloidal aspect. In particular, we will pay attention to the results on the associative properties of ASPs, which in turn affect the chemical composition and the thermo-mechanical performances of bitumen.

So far, several exhaustive reviews have appeared dealing with ASP aggregation by various techniques, each trying to illustrate the updated state of the research on the structure of bituminous materials. For example, in their review, Ghosh et al. [22] discussed ASP aggregation via spectroscopy methods, while Zhang et al. [23] focused on the relationship between microstructure and properties of bitumen, as highlighted by four microscopic testing approaches (FTIR, NMR, GPC, and AFM). Here, we will try to retrace in tutorial fashion the evolution of the salient results that over the years have gradually led to consolidating a colloidal model, with the aim of describing the aggregation behavior of ASPs in solution both in crude oils and in model solvents.

Other types of models that do not explicitly refer to concepts of the colloidal architecture of bitumen’s structure will also be recalled.

In this context, the review is subdivided into sections. Following this Introduction, Table 1 summarizes the principal achievements obtained in the study of the bitumen aggregation mechanism and classified according to two modeling approaches, i.e., solubility and colloidal. In the next Section (2), they are further illustrated and described in their essential features. Section 3 and Section 4 describe, respectively, experimental and computer simulation results reported on the study of bitumen microstructure and, finally, in Section 5, several current research challenges are briefly outlined. The last Section (6) features the main concluding remarks.

## 2. Asphaltenes and the Colloidal Nature of Bitumen Microstructure

**Figure 1:**
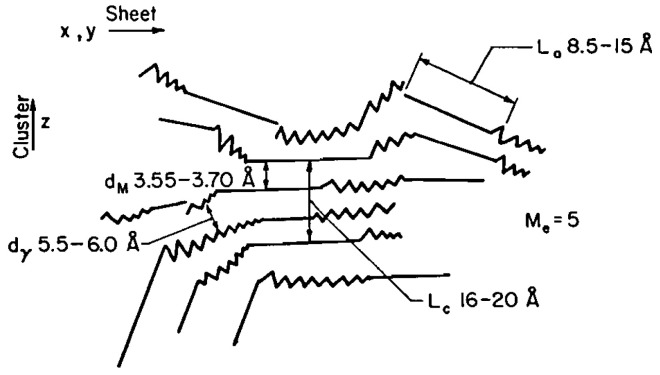
Dimensions of the proposed lamellar structure of asphaltene. Reproduced from ref. [32] with permission from ACS Publications.

The early models on bitumen microstructure borrowed the concept of “micelle” from colloid science, and suggested that ASP floats in suspension in the form of self-associated aggregates in a matrix of maltenes [24,25,26]. In its simplest form, this first model refers to bitumen as a colloidal system in which a suspension of ASP micelles is peptized by resins in an oily medium [27,28,29]. In particular, three different models of colloidal structure, namely, the solution sol, the elastic sol, and the gelatinous gel, were initially proposed [25]. Using these models, early petroleum scientists were able to explain the variations in the mechanical behavior of different types of bitumen, which have been observed experimentally. Since the early 1960s, Yen has been a pioneer in proposing a hierarchical scheme of ASP self-association, illustrating aggregation mechanisms ranging from the molecular state up to the cluster state [31,32]. Indeed, thanks to the first X-ray diffraction studies carried out on “pure” solid ASP, the initial version of the Yen model captured the stacking phenomenon of ASP, as illustrated in Figure 1.

ASPs, which have a strong tendency towards self-association, were considered capable of forming self-associated cores as the central unit of micellar-like supramolecular aggregates [32]. In particular, in the Yen model, ASP molecules were treated as plate-like structures capable of stacking together and forming a series of layers stabilized by various types of intermolecular interactions (π–π bonding, H-bonding and van der Walls interactions). It allowed us to gain an insight into the ASP macrostructure, such as the separation distance between two aromatic planes (d_M_), the aromatic layer diameter (L_a_), the height of the unit cell (L_c_) and the number of planes that contribute to the stacking of aromatic rings, and lastly, the distance between two neighboring aliphatic chains (d_γ_).

**Figure 2:**
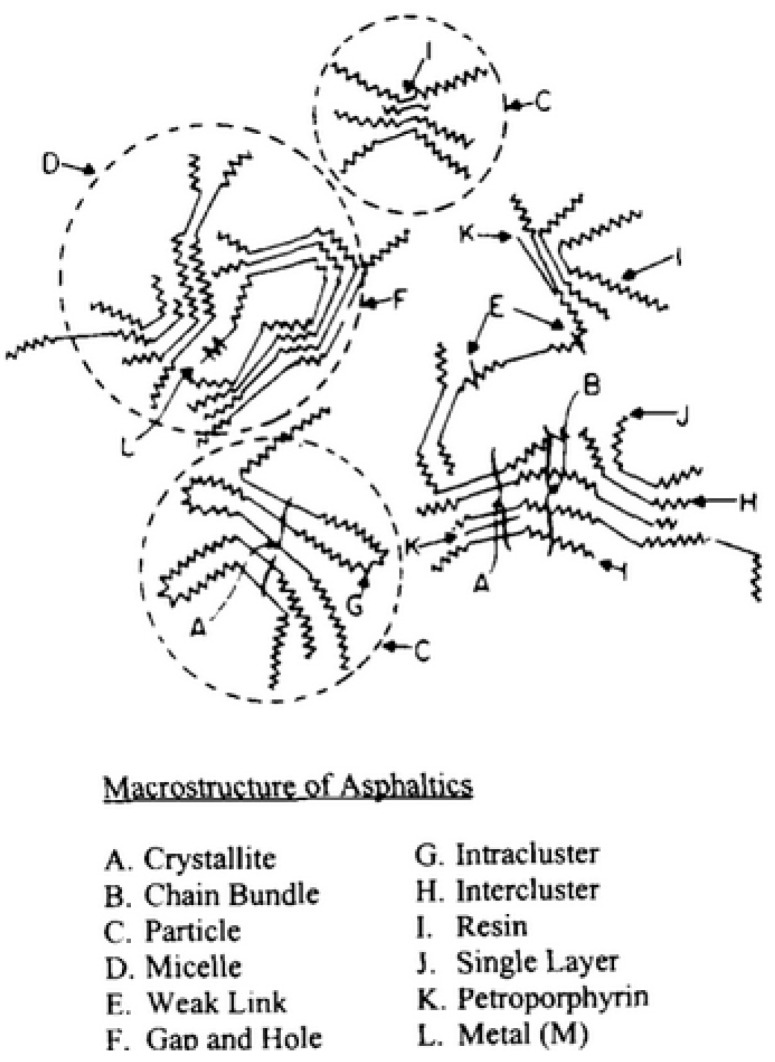
Hierarchical structure of asphaltenes at different length scales (reprinted from [34]).

In the micelle structure of the Yen model [32], bonds of sulfide, ether, aliphatic chain, and/or naphthenic rings constitute the bridge among other building rings. In this model, the micelle was said to be about 16–20 Å high and 8–16 Å wide. Yen’s proposed hierarchical view visualized the state of ASPs at different length scales focusing, in particular, on their propensity toward aggregation (Figure 2). The structure of the ASP molecule consists schematically of an aromatic region linked to aliphatic chains. The stacking phenomenon of several planar aromatic parts of the molecules leads to the formation of particles or crystallites, probably brought about by H-bonding or π–π association. Particles further associate into micelles, which in turn could form large aggregates. 

Then, some debate was launched about whether the micellar unit in petroleum is made up of a material of a homogenous nature, since it consists of only ASP molecules, or if it consists of ASP and resin molecules. An alternative point of view was proposed by Altegelt and Harle [35], who regarded bitumen as a solution of disaggregated ASP dissolved in maltenes as the solvent. Koots and Speight [36] inferred that resins or maltenes are fundamental, in that their role is to keep the ASP constituents in suspension, and therefore the micellar structure should be considered as an ASP–resin complex rather than an ASP–ASP micelle. Petersen [37] emphasized the importance of the different molecular components of bitumen interacting with one another so as to form a compatible or balanced system. Explaining the physical and chemical properties of bitumen using the solubility approach came to sometimes be known as the “Petersen model” [38]. Later, many investigations began to work around the mechanism(s) by which ASPs self-associate both in solution and in crude oil [57,58].

The proposal of an outright alternative compositional model and a direct confrontation to the colloidal model was put forward by Anderson et al. [39], who proposed the dispersed polar fluid (DPF) model, in which bitumen was envisioned as a fluid that consists of a continuous distribution of variably sized molecules and different polar functionalities. The associations between these molecules were attributed to H-bonding, π–π bonding, van der Waal bonds and polar–polar electrostatic interactions. The notable difference consists in the fact that while the colloidal one presumes the existence of a continuous, low-polar phase and a dispersed, highly polar phase [59], the DPF model assumes that bitumen is a single-phase system, i.e., a simple homogenous liquid [39]. However, the general acceptance of DPF by bitumen scientists as an alternative model to the colloidal one is still yet to be established.

**Figure 3:**
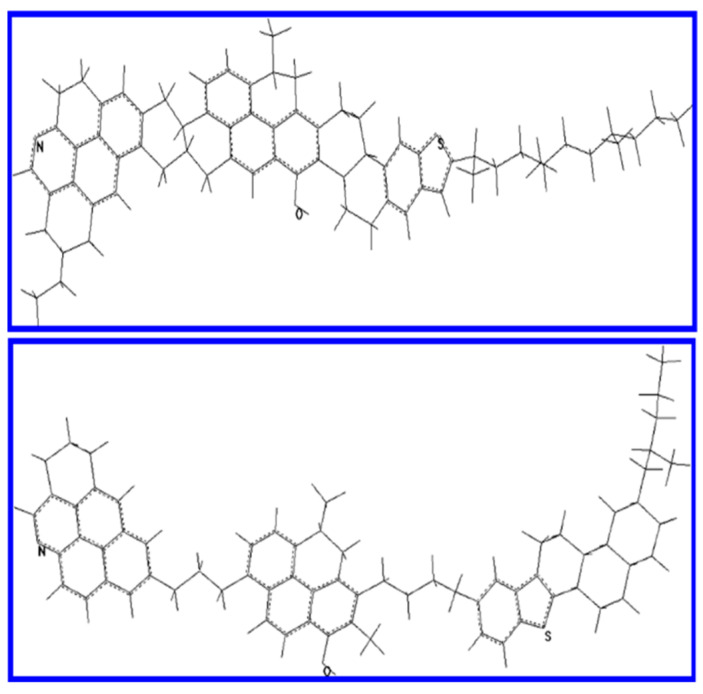
Condensed rigid structure of asphaltene for the insoluble fraction, A1 (above), and rosary-type flexible structure of an asphaltene model used to simulate compounds in the soluble fraction, A2 [44].

With the beginning of the 2000s, research in petroleum science focused on the effects of chemical structure and other components of bitumen on the aggregation mechanism of ASP [60,61]. A new model was proposed by Redelius [40], based on the mutual solubility of solvents. It incorporated information about cohesion, molecular volume, dispersive and polar interactions, as well as H-bonding. In particular, he developed a method to separately measure H-bonding and other polar interactions in aged asphalts and asphalt–flux mixtures. Concomitantly, the concept of micelles as the basic units of the colloidal model of bitumen continued to be tested in its validity. As it was observed by Lesueur [41], resins could play a surfactant-like role and stabilize ASP into micellar aggregates. Accordingly, the ASP micelles were visualized as consisting of an insoluble molecular core associated with resins, thus imparting steric stabilization that disfavors the flocculation and precipitation processes. The ASP phase’s separation after adding a non-polar solvent to the crude oil could thus be conceptualized in terms of reducing the polarity or the solubility parameter of the hydrocarbon. A thermodynamic equilibrium between individual ASP species and micelles was then invoked to explain ASP stability in hydrocarbon media and the stabilizing influence of the resins [42]. According to this model, the ASP colloidal particles were thus presumed to possess structures in the form of a core encompassed in a shell. The cores presumably form “insoluble” aggregates of stacked ASPs, which are stabilized by ASP molecules with a higher solubility [62]. This model agrees with the basic concept of Acevedo’s proposed description of the aggregation phenomenon [43]. In Acevedo’s rosary-type aggregate structure, the core of the particle is constituted by the least soluble fraction, A1, encompassed by a more soluble fraction, A2 [63,64], which functions as a solvating/dispersing agent [44] (see Figure 3). Studies involving the aggregation and dissociation of ASP solutions in maltene (solvent) showed that the colloidal aggregates could be increased by the addition of insoluble A1 ASPs, while on the other hand, the A2-type ASPs, which have a tendency to be more soluble, remained dissolved in maltene phase [45]. It has been indicated that the A1–A2 colloidal aggregates in this rosary-like structure are linked by the alkyl chains without necessarily being stacked via the π–π system. Even though the A1 monomer structure was rigid, the alkyl chain connection conferred flexibility to the rosary-type structures and a huge amount of both folded and unfolded conformers could be visualized [44].

**Figure 4:**
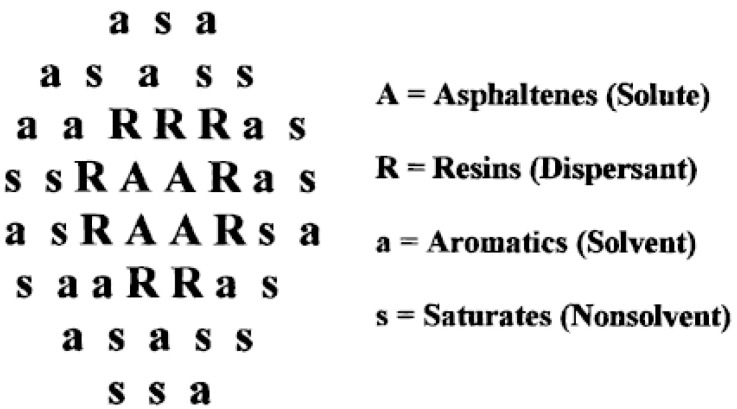
The Wiehe model of oil residue based on solubility [46].

Afterwards, to explain solubility and compatibility, a physical model of the oil residue was proposed by Wiehe et al. [46], which was believed to be an aggregation phenomenon as a result of the changes in solubility. This proposed micelle structure was the backbone of the colloidal model. In this model, the resin molecules (R) form a layer around the ASP core (A), which in turn is further covered by a subsequent layer of aromatic molecules (a), all enveloped by an outer shell consisting of saturated substances (s) (see Figure 4).

In the context of the colloidal model, the classification of bitumen in terms of its ASP content is mentioned here to differentiate low-ASP (sol-type) binders (which are ductile, have low degrees of complex flow and show large variations in viscosity with temperature) from gel-type binders, with a high ASP content and characterized by non-Newtonian rheological behavior, relatively low viscosity variation with temperature, low ductility and susceptibility to hardening by oxidative aging [41]. Bitumen with intermediate ASP content has properties intermediate between the extremes of sol- and gel-type behavior. The transition from sol- or sol–gel- to gel-type materials that accompanies the increase in ASP content has been postulated by many researchers as the cause for bitumen failure by embrittlement and cracking [65].

Then, two questions began to arise: what forces are behind the association of the ASP molecules and at what point does aggregation result in precipitation? The forces behind the association phenomenon somehow need to have a greater magnitude than those that maintain ASPs in solution. As a matter of fact, aggregation and precipitation are driven by different intermolecular forces: strong forces instigate association, while precipitation occurs as a result of dispersive forces among the aggregates. The ASP flocculation phenomenon could be instigated by solubility changes (the addition of a paraffinic antisolvent). However, both questions could not be analyzed within the framework of the early version of the Yen model, as it suffered from severe setbacks. It was inferred from investigations on ASP (solid) powder, and it was not firmly proven, that the model held also in solution. Thus, the representation of the elementary units of micelles was modified by contemplating a further clustering into particles of larger sizes, which bear similarities to the physical structure of a flake [47]. The picture consisted of various combinations of unit sheets of molecules forming particles, micelles, and clusters of micelles with different mechanisms of molecular interaction.

**Figure 5:**
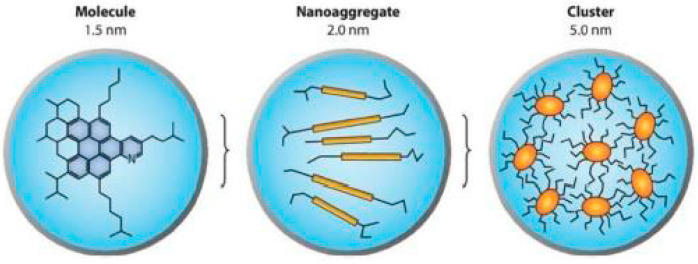
Yen–Mullins model of the asphaltene molecule, nanoaggregate (particle) and cluster [49].

Thus, the Yen model was subsequently modified by Mullins [34,48,49], which distinguished several different entities (Figure 5): the elementary molecule, the nanoaggregate particle, and the cluster of nanoaggregates. In the Yen–Mullins model, an ASP molecule is in the range of ~1.5 nm; a nanoaggregate consists of stacked aromatics with a small aggregation number (<10) and a size of ~2 nm, while higher concentrations form clusters of nanoaggregates, again with small aggregation numbers, starting at ~5 nm [50]. These different entities may be associated by several types of interaction. ASP could precipitate artificially or naturally if molecular interactions with resin constituents are disrupted [66].

Other NMR results were also found to be consistent and in concordance with the hierarchical Yen–Mullins model [67].

### 2.1. Aggregation of Asphaltenes in Bitumen and Model Solvents

The aggregation behaviour of ASP is plausibly influenced by the chemical composition, polydispersity and steric arrangement, or the interconnective network of functional groups in the single ASP monomers. This complex mixture is usually in a delicate equilibrium, which can be modified by a change in the temperature, pressure, or composition, resulting in asphalt precipitation [68]. Irrespective of the geographical origin and physical properties of ASPs, the calorimetric data showed that ASP aggregates form a low-cohesion energy, polydispersed system [69]. Filtration has demonstrated size polydispersity, since varying quantities could be recovered by using varying pore size membranes [70]. The aggregation rate and equilibrium of aggregate dissociation are influenced by concentration, temperature and time. Van der Waals forces are dominant in concentrated solutions, while coulombic forces are largely responsible for aggregation in diluted solutions [71].

Hence, a deep understanding of the mechanism of the association phenomena is critical for determining the true molecular parameters (weight and size), and also for developing solutions to the problems brought about by ASP, as highlighted in the Introduction. A wide array of theoretical and experimental techniques has been applied for the study of such complexity. Unfortunately, the understanding and description of the structures of these complex fluids is still challenging and difficult due to the enormous number of their constituents. A simplified approach consists in the extraction of ASP from these fluids and its consequent dissolution in pure organic solvents, such as toluene, in order to access the structural parameters. However, it is worth noting that the observed behaviors in model solutions do not always have to reflect the actual state of crude oil, much less live crude oil. ASP particles in oil may be partially dissolved, partially micellar, and/or partially (steric) colloidal, depending on the polarity of the milieu. The addition of resins to ASP solutions was initially interpreted in terms of the effect of improving H-bonding due to specific interactions between resins and ASPs [72]. Some part of the ASP fraction would be prone to associate and aggregate even in the best solvents, such as toluene. In dilute solutions, the concentration limit, which has been observed for the self-assembly of ASP monomers, was reported to be less than 10 mg/L [62]. Stacking as an inceptive mode of association might come about by the interaction of π-orbitals via complexes of charge transfer of the aromatic rings, as shown by the results of early X-ray diffraction analyses [33]. Stacking involves five to six layers of molecules, but only two or three according to, respectively, refs. [33,73]. Stacking via interactions in the aromatic core was hypothesized to happen between colloidal particles as well [62].

According to Creek [74], the forces that influence precipitation and aggregation are the factors that differentiate every single one of these processes. He also differentiated aggregation from precipitation as definite steps in a process that is totally reversible. Strong interaction sites, which are found at the edges of the ASP molecules, promote aggregation phenomena, beginning with their reversible association in 2D sheets (lamellar array). Meanwhile, influenced by van der Waals forces of attraction between aggregates, precipitation eventually may occur [74]. Considering the arrangement of the ASP molecules in the aggregates, in principle, it should be different from a surfactant system, partially because of the inhomogeneity of the structures of the ASP molecules. How are ASP molecules arranged in an organic solvent in order to form aggregates, and what is the principal difference between the aggregate surface and its core? These are two fundamental questions that still have not been answered. Although there is some understanding about colloidal structures, the underlying mechanism of the packing of ASP molecules in an aggregate is as yet unknown.

### 2.2. Reversible Aging and Thermal Behavior of Bitumen

One of the typical phenomena occurring at low temperatures in bituminous materials under isothermal conditions is the thermoreversible aging caused by physical interactions, such as crystallization, phase separation and vitrification, all of which being reversible upon heating to sufficiently high temperatures [75]. Reversible aging can be divided into steric hardening, which occurs mainly in the intermediate temperature range, and physical hardening, which usually refers to a reversible phenomenon at low temperatures. Since the low-temperature properties of bitumen have important effects on pavement cracking performance, understanding these processes is crucial in evaluating the effects of physical hardening in both laboratory testing and field studies. Bitumen, as a viscoelastic material, is generally able to endure significant thermal stresses occurring under various cooling conditions. However, the ability to relax stress decreases when temperatures that are too low make the binder very stiff, and consequently there could be a risk that transverse cracks may occur throughout the entire paved section. Unlike oxidative aging associated with oxidation processes and chain breaking in organic polymers, physical aging does not imply chemical change, and represents a reversible phenomenon of the amorphous glassy state. Indeed, heating the bituminous material erases the effects of physical aging, and the memory of its previous physical state is completely lost.

Bahia and Anderson studied physical hardening and glass transition in bituminous binders characterized by good and poor low-temperature performance [76]. A clear correlation was found between the physical hardening and glass transition process and the non-equilibrium state of bitumen in the range around the glass transition temperature (T_g_). Anderson and Marasteanu illustrated several methods for describing the changes in viscoelastic moduli that result from physical hardening [77]. It was postulated that the physical hardening mechanism did not correspond to that typical of polymeric systems, and that this discrepancy could be attributable to the crystallizable fraction (waxes, paraffins, saturates) present in bitumen. The combined effect of both free volume collapse and wax crystallization was invoked to explain why isothermal hardening occurred at temperatures well above the T_g_. Masson et al. [78] conducted a systematic investigation to correlate the physical hardening phenomenon with the various SARA fractions constituting the crude oils, and concluded that while both ASPs and resins made the main contribution to the hardening effect, maltenes could manifest a significant effect over the kinetic aspect of the whole process. The effects of the crystallization of waxes and internal rearrangements of ASP were recognized by Hesp et al. [79] as possible mechanisms to explain reversible aging. By using AFM and neutron scattering techniques, Schmets et al. [80] found that gradual phase separation at low temperatures was triggered by the nucleation of wax crystals, and a new hypothesis was developed for its effect on the cracking and healing potential of bitumen. Tabatabaee and coworkers [81] proposed a prediction model, based on a modified creep viscoelastic model, capable of describing the rate of physical hardening as a continuous function of time and temperature. 

Fischer et al. [82] investigated the kinetics of the crystallization process of bitumen by observing with AFM the development of the *peri*-phase structures, which in turn was connected with an increase in stiffness and hardness. Isothermal DSC measurements were also carried out, and the results were analyzed to interpret the observed AFM images.

Laukkanen et al. [83] investigated the low-temperature mechanical properties of bitumen and considered the effect of reversible aging on the shape of the binder relaxation spectrum. They used the complex glass-forming liquids theory to explain the coexistence of liquid and glassy microphases in bitumen when annealed at low temperatures. Ding and coauthors [84,85] compared the low-temperature reversible aging discrepancy mechanism for binders with similar low-temperature performance grades, and evaluated the effects of the contribution of various pure waxes added to model bituminous materials. Another work compared the effects of both types of hardening, namely, oxidative (irreversible) and thermoreversible aging, on the performance grade parameters, such as complex shear modulus, the elastic and viscous components of the complex shear modulus, and phase angle [86]. The authors analyzed the calorimetric data under the Ozawa theoretical framework, which has proven to be an effective method of predicting the rate of transition from an amorphous to a semi-crystalline state during isothermal annealing.

### 2.3. Critical Micelle, Critical Nanoaggregation and Critical Cluster Concentrations (CMC, CNAC, CCC)

In solution, ASP micellization was postulated very early [25] as a structural model of bitumen. An unanswered question is about the forces that take part in the mechanism of micellization, which comprise π–π interactions between aromatic nuclei, dipolar and charge-transfer interactions, as well as van der Waals interactions and H-bonding [87]. The π–π interaction was identified as the type of interaction that brings about micelle formation, and was hypothesized to involve heteroatomic moieties [51]. Critical micelle concentration (CMC) or critical aggregation concentration (CAC) was proposed as a prelude to agglomeration to establish the concentration value at which the aggregation of ASP begins [88]. In light of this, the resins added to the ASP solvent medium take part in micelle formation, and are not involved as cosolvents. Fourier transform infrared (FTIR) spectroscopy was employed to study the primary contributor to micellization in ASP solutions from three Chinese VRs produced in the oilfield of Liaohe, Gudao and Shengli. Liu et al. [52] indicated that the H-bond was the main association force between ASP molecules; π–π interaction, electrostatic forces, as well as polarity induction were also implicated as contributors to the association phenomenon.

An alternative concept was derived from the fact that ASP molecules are in a medium with a much smaller average MW. In view of this, ASP and oil represent a true solution, and their colloidal behavior is due to the huge difference in dimensions between ASP and oil (lyophilic colloid). This led to a description of the behavior of the system through the traditional thermodynamic equations for liquid–liquid equilibrium, thus avoiding the presupposition of a colloid [53].

At concentrations lower than CMC, molecular association mostly occurs. Several studies have shown that below the CMC range, association phenomena between ASPs could begin. This additional evidence gave more validity, credibility and coherence to a gradual associative mechanism than one that assumes the formation of finite-size micelles and the existence of CMC [89,90,91]. In aromatic solvents, at concentrations higher than CMC, the aggregates would derive from ASP particles, and the aggregation behavior would only be influenced by aggregate diffusion. Above the CMC, the aggregation of micelles occurs whose dimensions are independent of the increasing concentration of ASP [92]. On the other hand, the mechanism of micellar association via coacervation was hypothesized by Priyanto et al. [93], who assumed values greater than 25 nm for the size of the aggregates. Micellar particle formation has also been reported by other authors [94].

As the ASP concentration gets higher, the micelles tend to get bigger, and there is an increase in the potential barrier as a result [95]. The concentration effect has been simplified based on the classic Derjaguin, Landau, Verwey, and Overbeek (DLVO) theory, which implies that two factors affect the kinetics during the aggregation of colloidal particles: diffusion and reaction [96].

Several reports have claimed that the CMC can be evaluated via several methods, such as surface/interfacial tension, calorimetric titration, or spectroscopy. The CMC of ASP determined with these methods ranged from 2 to 18 g/L for different crude oils [89,97,98]. Sheu et al. [99,100] used surface tension measurements to evaluate the CMC of ASP extracted from Ratawi vacuum residue in pyridine, and found a value of ~0.4 g/L. Other studies involving surface tension measurements have reported higher CMC values for Brazilian ASP (10 g/L) [101] and Algerian ASP (1.7 g/L) [102], both values referring to the toluene as solvent. Using the same technique, Rogel et al. [103] reported that the CMC values of unstable ASP in different solvents varied in the range of 1–30 g/L. Larbi et al. [104] estimated the CMC of two Algerian ASPs dispersed in toluene, pyridine, and nitrobenzene solvents, and found values between 0.98 and 4.17 g/L. They concluded that the aggregation behavior was principally driven by the structure and polarity of the ASP, as well as by the solvent polarity.

**Figure 6:**
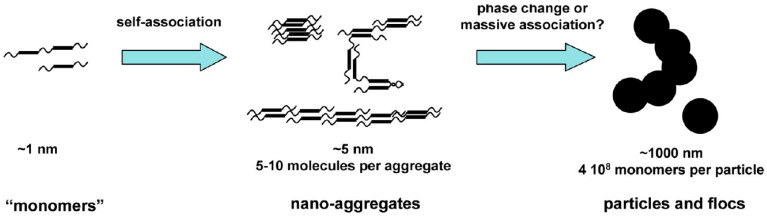
Yarranton’s model of the oligomer or supramolecular association of asphaltenes [55].

Surface tension and vapor pressure osmometry (VPO) measurements of ASP solutions of increasing concentration indicated a rise in the number of median MW, which was linked with a higher degree of association until a limiting value was reached [105]. However, the observed linear decline in interfacial tension with respect to ASP concentration showed that micelle formation can be ruled out, and that the aggregation observed with the VPO technique is not correlated with the micellization mechanism. Based on the above experimental evidence, in order to describe ASP association, Agrawala and Yarranton [54] proposed a stepwise polymerization model, which was modeled analogously to linear polymerization (see Figure 6). The principal concept was that ASP molecules may possess one or more active sites (functional groups), which are able to link and interact with the other ASP units. For example, oxygen and nitrogen active sites (functional groups) bring a higher polarity to the molecules, activating participation in strong intermolecular associations. Carboxylic acids, carbonyls, phenols, pyroles, and pyridines that are capable of taking part in proton or donor-acceptor interactions have also been recognized in ASP [106].

ASP consists mostly of propagators, but at the same time contains a small fraction of terminators. Analogously, resins consist mostly of terminators, but at the same time contain a small fraction of propagators. Molecules having more than one active site play the role of propagators, and molecules with only one active site play the role of terminators in the association process. The strength and nature of the potential links may be considerably different due to the fact that ASPs comprise thousands of chemical species, which feature a wide range of functional groups.

Other surface tension measurements [107] and micro-calorimetric studies [97] confirmed the prediction of the ASP structure at several length scales, as proposed by the Yen–Mullins structural model [48]. It is worth noting that with low aggregation numbers (N < 10), the critical threshold of the transition from monomers to nanoaggregates is not so sharp in terms of concentration [108]. Nonetheless, several researchers have preferred to make use of the term “critical”, and define the CNAC as the concentration whereat the further growth of individual nanoaggregates ceases.

High-Q ultrasonic studies were the first approach utilized to effectively determine the CNAC of ASP in toluene, with values in the range ~50–150 mg/L, depending both on the type of ASP and the solvent [109,110]. According to direct current (DC) electrical conductivity measurements performed at concentrations both lower and higher than CNAC, it was found that the ASP nanoaggregates initiated their formation at very low concentrations in toluene solution, and their aggregation number was <10, with the nanoaggregate size on the order of 2 nm [5,111,112]. Goual et al. [113] established an outstanding agreement between the CNACs evaluated by the centrifugation and DC electrical conductivity methods, obtaining a value of 150 mg/L for ASPs extracted from different crude oil sources.

Besides the primary ASP aggregation of nanoaggregates, a secondary process of the formation of clusters known as the critical cluster concentration (CCC) occurs at a mass fraction slightly above 10^−3^, as has been assessed by both AC-conductivity [114] and DC-conductivity measurements [112,113], corresponding to concentrations of about 4 g/L [115,116]. Low-field ^1^H-NMR results showed the relaxation enhancement of crude oil by the transitory entanglement of solvent hydrocarbons within ASP clusters, and this led to the proposal of a porous ASP model, which was in line with the experimental data [117].

Thus, the formation of nanoclusters (or nanoaggregates) occurs as a result of molecular association, and this phenomenon has been observed in toluene solutions, dead oils, live oils, and bitumen [118,119,120,121,122,123]. Be that as it may, these nanostructures, representing only a small part of the entire amount of ASPs, may still be stable, and as stated by previous studies [119,124], they would not be increased by association with other molecules around them. In addition, the alkyl substituents present in the aromatic rings of ASP molecules might cause a steric inhibition for stacking [125]. As a matter of fact, steric effects could hinder the growth of aggregates and influence their size, although for those inhibited molecules, other forces may preside over these, and a different mechanism of association would occur. Though steric effects might inhibit stacking [126], π-orbital interaction remains a logical and valid mechanism for association. The forces that exist between molecules that prevail over the formation of aggregates in ASP have been enumerated by Murgich [127] as: (i) intermolecular charge transfer; (ii) short-range exchange repulsion energy; (iii) weak inductive interaction; (iv) electrostatic (coulombic) interaction between the molecular charges; (v) van der Waals interaction. Additionally, it is worth noting that van der Waals interactions occurring in a certain aggregate do not prevent or hinder the action of another force from acting on other fragments of the molecules.

**Figure 7:**
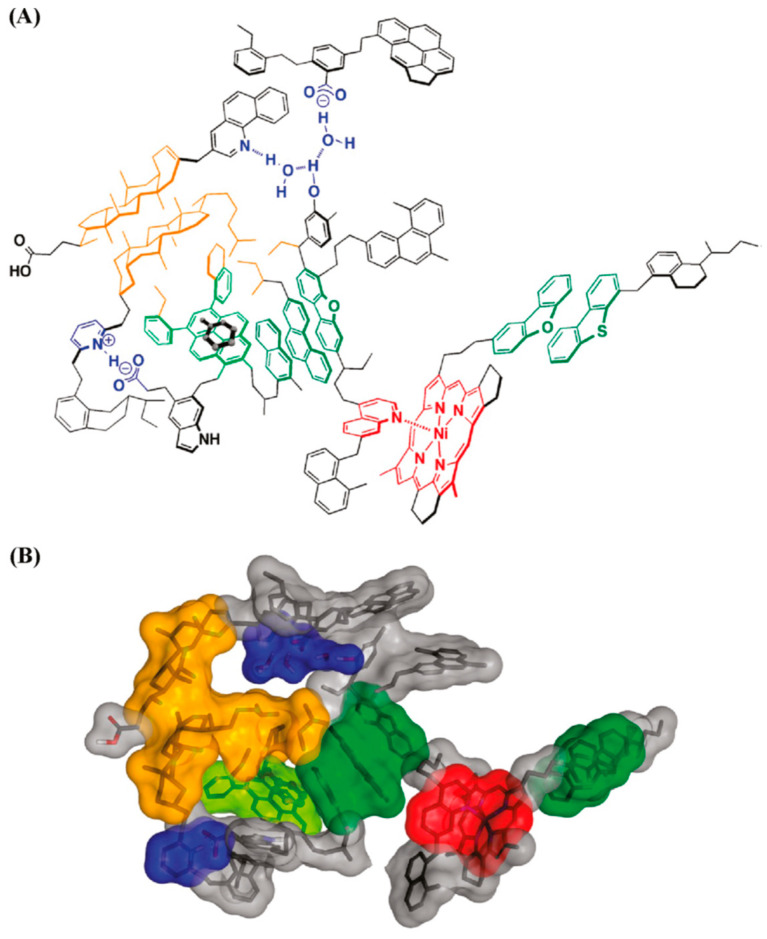
Gray’s model of a supramolecular assembly in a representative asphaltene aggregate. Associations between molecules are color-coded in (**A**) the molecular depiction and (**B**) the space-filling version: acid–base interactions and hydrogen bonding (blue), metal coordination complex (red), a hydrophobic pocket (orange), π–π stacking (face-to-face dark green; within a clathrate containing toluene, light green) [56].

Both the Yen and Yen–Mullins models emphasize the formation of molecular clusters via the π–π stacking of aromatic rings. Subsequently, a supramolecular assembly model for ASP aggregation whose driving forces comprised acid–base interaction, H-bonding, metal coordination, hydrophobic pockets, as well as π–π stacking was proposed by Gray et al. [56]. The supramolecular model included a selection of types of molecular structures and architectures, which the authors stated may coexist in bitumen. This supramolecular assembly of complex molecules, illustrated in Figure 7, implies that the ASP nanoaggregates dispersed in solution could generate structures with pores and host–guest complexes, such as, e.g., organic clathrates, and in this case, occluded guest molecules would play the role of stabilizing the assembly of a cage.

At the supramolecular length scale, differing points of view exist regarding, in particular, the properties and behaviors of the self-assembling of the ASP fraction. This division of opinion comes about partly as a result of the uncertainty of the molecular and submolecular structures. Several different molecular prototypes are envisioned [128] using the concept of CMC typical of colloidal solutions [103], and as a result, several different mechanisms of self-association have been identified and proposed [33,55,56,129]. These mechanisms entail the assembly of dense and open supramolecular or colloidal structures, which are kept together by noncovalent bonds such as acid–base interactions, H-bonding, hydrophobic pockets and aromatic π–π stacking. Another reason behind the differing opinions at this length scale is the uncertainty of the definition of ASP. In addition, the link existing between the chemically separated ASP fractions and the nanodispersed molecular species in crude oils seems rather weak [119,130]. With these caveats in mind, a lot of the research attempted on the nanometer length scale can be divided into two totally different and irreconcilable lines of interpretation. In one of these lines, ASP nanoaggregates are thought to be soluble in crude oil and toluene, and assemble, destabilize or precipitate from the solution upon the addition of n-alkanes or by varying intensive variables, such as pressure and temperature. Changes in pressure shift solvent-like materials either in or out of the oil phase, and as such, greatly influence the oil stability; as a result, ASP precipitation also gets affected. On the other hand, along the other line, ASP nanoaggregates are thought to be dispersed and not solubilized, either in crude oils or in toluene. The observed behaviors are then interpreted as a change in the type or magnitude of aggregation. Most of the physicochemical properties of bitumen can be described using the conventional viewpoint, including phase behavior (using regular solution theory and equations of state) and physical properties (e.g., viscosity correlations based on Newtonian flow). However, cases exist in which the conventional approach is not logical, and these drawbacks have begun to be systematically probed. For instance, bitumen displays non-Newtonian behavior at temperatures below 300 K, stipulating the formation of structures in the fluid [131].

## 3. Experimental Investigations

Various techniques have been employed in characterizing the microstructure of bitumen. One of the main challenges of data interpretation arises from the pronounced polydispersity of ASP suspended in solution, and this is apparently reflected in the wide range of MWs that have been experimentally determined using different measurement methods.

### 3.1. Atomic Force Microscopy (AFM)

The Atomic Force Microscopy (AFM) technique performs a scan of the surface of a test sample by “feeling” its surface using a sharp and usually pointy conical or spherical tip attached to the end of a cantilever. The apex of the tip has a size of a few nanometers, and the cantilever is about 1 or 2 mm. A surface profile can be obtained from the movement of the tip over the test surface by evaluating the frictional and attraction forces between the surface and the tip. The measurement of the frictional and attraction forces generates phase images, which highlight the diversities on the surface of the sample being tested. The spatial resolution of conventional AFM images is about a few nanometers over an image size of approximately 25 × 25 to 50 × 50 μm. The study that perhaps first observed and identified two or more phases existing in bitumen via AFM was carried out by Loeber et al. [132]. They referred to the discrete phases as “bee structures” due to their peculiar shape. In more comprehensive terms, the typical phases observed in bitumen via AFM may be referred to as the matrix, the inclusion, and the matrix–inclusion interface, or in a general sense, are known as microdomains. Specifically, from the image analysis, bitumen’s morphology is classified into four phases: *para*, *sal*, *peri*, and *catana* (bee structure). This subdivision is useful in studies on the correlation between the degree of aging of the bitumen and its microstructural characteristics, in particular the change in the bee structure. Subsequently, a few research groups, such as Allen et al. [133] and Jahangir et al. [134], have identified bee structures having average heights of between 22 and 85 nm, and typical distances between ridges of the bee-backs of approximately 150 nm. Masson et al. [135] proposed a connection between the bee phase and ASP, while other research studies have pinpointed bee structures as crystalline wax structures [136]. A comprehensive survey of the results reported on the application of AFM to the analysis of bee structures and how they are affected by the oxidation of bitumen can be found in the recent review by Zhang et al. [23].

### 3.2. Scattering Techniques and Fractal Nature of Bitumen Microsctructure

Several direct and indirect spectroscopic methods exist, some of which include small angle neutron scattering (SANS), small angle X-ray scattering (SAXS) and X-ray diffraction (XRD). The aforementioned spectroscopic techniques are the most suitable due to their compatibility with colloidal systems from the resolution and length scale point of view.

SAXS and SANS are techniques that have been used for a long time in the elucidation of the microstructure of the colloidal units constituting the bituminous materials [137,138]. Both of these techniques are easily accessible, are optimal in resolution, and their mechanical theories of statistics used for data analysis are mature. Another important factor is that both of these techniques can be used to carry out in situ measurements, and this opens up the door for the possibility of carrying out a kinetic study. Apart from structural studies, these techniques enable the investigation of other characteristics of ASP, such as its (1) surface, (2) interfacial, (3) short-range bulk and (4) long-range bulk properties.

SAXS provides a topological view of the ASP aggregate systems that scatter the X-rays via the ASP’s electron density differences from one molecule to another, as a result of their medium and polynuclear aromaticity. Thus, it can be said that SAXS is a suitable technique for carrying out studies on ASPs in their natural environment. In SANS, scattering occurs in all the nuclei, and consequently the technique is not used to study ASP in their natural state. However, this technique has proven to be useful for studying ASPs dispersed in solvents. For example, results from Lesueur’s work [41] with SAXS and SANS were used to corroborate the colloidal model, while Redelius [139] proposed that the results obtained from the application of these techniques can only be interpreted according to predetermined models. The contrast between the surrounding fluids and the ASP can be increased by using perdeuterated solvents. This is brought about by the significant differences in the scattering lengths of protons and deuterons.

Fractal theory provides a powerful quantitative framework for describing the properties of complex systems and processes observed in nature [140]. The fractal dimension D*_f_* of an aggregate can give key insight into its morphology (e.g., compact versus loose), as it describes aggregates shaped by the association of very small unit particles. Thus, various investigations mainly derived from scattering measurements have exploited the fractal model to analyze both the bitumen microstructure and ASP model solutions in aromatic solvent. Liu et al. [141] and Barré et al. [142] found aggregates having a fractal-like structure in concentrated ASP suspensions in n-heptane. By using the photon correlation spectroscopy technique, it has been found that ASP flocs can reach sizes in the range of 4–5 μm when ultimate sedimentation occurs [143], and this phenomenon is corroborated by fractal-like diffusion-limited aggregation [144,145,146,147].

Under shaking, unstable fractal aggregates were dissociated, and this brought about the formation of basic aggregated particles with average sizes of the order of 1 μm. The aggregation–flocculation kinetics of ASP are categorized into three stages. The first stage involves a nucleation process that implies the formation of ASP clusters of critical size. In the second stage, the clusters absorb either small-sized micelles or ASP molecules from the solution, resulting in a cluster growth. Finally, the third phase concerns the basic aggregates that merge to form fractal structures [143].

Later, Eyssautier et al. [130,148] analyzed hierarchical ASP aggregation up to the mesoscale from SAXS and WAXS measurements. Thus, ASP molecules have been found to form nanoaggregates able to assemble into fractal clusters in a further aggregation step.

A descriptive model of ASP association and precipitation was developed by Porte et al. [149], who proposed that the stacked 2D layers could lead to the formation of very flexible monomolecular sheets. The spontaneous folding out of the aromatic plane would in turn lead to the formation of hollow spherical vesicles. The high polar and less soluble fraction of bituminous compounds (ASP) was found to be susceptible to aggregation, forming sparingly soluble agglomerates. The aggregates obtained from the less soluble subfraction were related to the most common interaction forces, such as π–π interaction, H-bonding and electron donor–acceptor interactions between the aromatic and polar moieties. In the absence of solvating resins, the fractal dimensions of the aggregates were in the range of D*_f_* = 1.7–2.1, while in the presence of resins, the compactness of those structures increased by D*_f_*~3. Molecularly speaking, ASP molecules with low solubility were presumed to be of the “archipelago” type [150,151]. In addition to the “island” and “archipelago” designations of molecular architecture, Schuler et al. [152] have recently proposed a third class of molecular arrangement, defining it as having a single core consisting of aromatic carbons and containing one or more aryl linkages between aromatic moieties. This group of structures was definitively revealed by AFM imaging measurements [153,154]. According to the authors, the new classification “aryl-linked core” should reduce confusion between both “island” and “archipelago” structures, and help sort out the relationships between structure and function, especially concerning aggregation and reactivity.

Regarding the structural information extracted from scattering techniques applied to diluted ASP solutions, two approaches are generally pursued. One considers the Guinier and Zimm approximations valid in the analysis of the low-angle region of spectra for calculating the radius of gyration (R_g_) and MW. In studies that used the Ornstein–Zernike model to fit the scattering data, the reported values for R_g_ were found between 40 and 60 Å, and MW was approximately 50 kDa [144,145,146,155,156,157,158]. Combining viscosity and scattering experiments, the fractal model could be used to characterize the structure of ASP aggregates in good solvents, which as a result could be partly trapped [159].

Another method consists in the analysis of the experimental SAXS and SANS spectra in the whole *q* range, and assuming a priori the particles’ shapes in the fitting procedure of form factors. However, this method, which is model-dependent, has significant flaws, introduced first by the choice of both the polydispersity function and the presumed form factor, and second by the large amount of adjustable parameters [137,142]. Moreover, it is worth noting that SAXS and SANS patterns are practically superimposed at low *q* values, indicating that ASPs are homogeneous objects at large length scales, while this does not hold in the high *q* range, where a smooth power law observed in the X-ray spectrum is distorted in the neutron spectrum by the presence of oscillations, which is typical of a fine structure [138].

So far, numerous SAXS and SANS studies have been used to probe the nanoscale organization of ASP fractal aggregates in both model systems [55,122,141,144,145,146,148,155,157,158,159,160,161,162] and crude heavy oils (vacuum residua) [163,164,165,166,167,168]. Owing to the low scattering contrast between aggregates and maltenes, most measurements were reported for ASP in toluene and at different temperatures at which the model solvent improves the scattering contrast. In particular, it is difficult to apply SANS to real crude oils as a result of the high disjointed and somewhat unclear scattering cross-section of hydrogen, whereas SAXS has the potential to distinguish ASP aggregates’ structures in real systems. However, the general behavior of ASP in maltenes is quite similar to ASP in toluene. A method of direct analysis of ASP aggregation in crude oils was suggested by Tuzikov et al. [169], who analyzed the SAXS spectra of particles dispersed in selected types of crude oils to determine the structure and shape, and to calculate their size distribution. They attributed the fraction of 0.8–2.5 nm to resins, whereas larger aggregated particles with sizes up to 8 nm were ascribed to ASP. It is worth remarking that SAXS is a technique that can be used to study ASPs in their natural state (for a recent and comprehensive review on the applications of this technique to bituminous materials, see ref. [170]). SAXS provides direct information on the structure, dispersion composition, and mutual distribution of scattering particles in the sample. The method makes it possible to determine the size and shape of individual ASP molecules, as well as their aggregates, the sizes of which can reach 100 nm. Early scattering studies assessed by Dwiggins Jr. confirmed the colloidal structure of crude oil [171]. Pollack et al. [172] analyzed the SAXS profiles of powdered samples using the Porod treatment for closely packed systems, while R_g_ values in the range 30–70 Å for ASP suspensions in organic solvent media were determined with Guinier’s method. In a further and more accurate SAXS study, Dwiggin Jr. concluded that [173]: (1) Some crude oils can contain up to three different types of colloids (three peaks were found in the SAXS spectrum), and these colloids can respond to heating and solvent addition in different manners. (2) Some molecules used to increase crude oil production, mainly aliphatic straight and branched-chain hydrocarbons and alcohols, can produce colloid growth, while others such as cyclic aliphatic, aromatic, and aliphatic-substituted cyclic aliphatic and aromatic hydrocarbons do not. However, for those experiments carried out in the solid phase, ASP self-association in petroleum liquids was still unclear. 

ASP aggregates 3.4 Å thick and with radii in the range 13–800 Å were derived by fitting the observed scattering intensity with a model of disk-shaped particles [174,175,176], while there appears to be general unanimity on their high polydispersity. R_g_ values of 33-252 Å for Safaniya ASP aggregates in toluene were obtained from SAXS experiments upon fractionation by ultracentrifugation [157]. Savvidis et al. [70] carried out SAXS measurements of ASP in toluene solutions at room temperature, and discovered aggregates that had an R_g_ value of about 60 Å. On the other hand, huge-sized aggregates of the order of 1 μm were also observed via SAXS measurements of ASP powder carried out at room temperature, and these results were confirmed through scanning electron microscopy (SEM) measurements. Espinat et al. [177] performed SAXS experiments on dilute solutions of ASP in toluene to investigate the effect of a temperature decrease from 303 K to 263 K on the aggregation behavior. Indeed, a strong aggregation, correlated with the generation of fractal-like structures (D*_f_* = 2.05 ± 0.15), was observed upon decreasing the temperature. A different interpretation of the scattering data was provided by Sirota et al. [178,179,180], who interpreted the SANS and SAXS data in terms of short-lived density fluctuations of the ASP dispersed in solution. The fractal appearance of precipitated ASPs is a result of their phase separation into a very viscous glass-like state. The high viscosity of bitumen and ASP mixtures, which is also temperature-dependent, was then associated with their closeness to glass transition.

**Figure 8:**
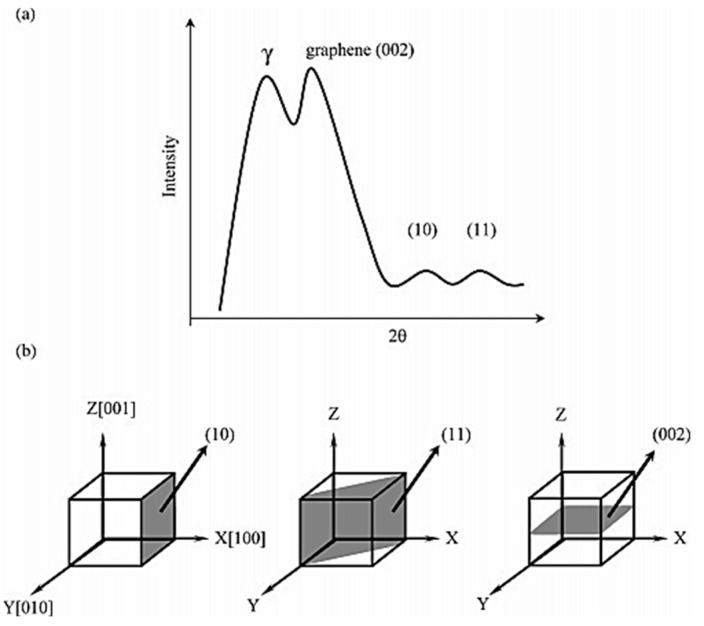
(**a**) Schematic of X-ray diffraction pattern and (**b**) diffraction planes in asphaltene structures. Reprinted with permission from ref. [181] 2002 American Chemical Society.

The complementary XRD technique provides information on the internal structure and crystallite parameters relevant to the molecules, which are associated in the aggregates. Typically, four peaks can be observed in the powder XRD spectrum acquired for bitumen, as illustrated in Figure 8; namely, the γ-band related to the aliphatic interchain distance d_γ_, graphene or (002) band, which corresponds to the spacing distance d_M_ between two aromatic sheets [169], and a pair of low-intensity bands ((10) and (11)). Starting with the diffraction angle θ corresponding to, respectively, the maximum γ-band and the (002) band, the Bragg relation $d=\frac{k\lambda }{2\mathrm{sen}\theta }$ is used to estimate either d_γ_ (k = 5/4) or d_M_ (k = 1), where λ is the wavelength of the Cu Kα radiation used as the X-ray source. The average layer diameter of the aromatic sheets between 7 and 10 Å and the average distance between aromatic sheets between 3.5 and 3.7 Å were derived from XRD experiments on the Ratawi and Kuwaiti ASPs [181].

The percentage of carbon atoms in aromatic structures, defined as aromaticity *f*_a_ [182], can be derived from the areas of the (002) and γ bands in the XRD pattern according to the formula *f*_a_ = C_A_/(C_A_ + C_S_) = A_(002)_/(A_(002)_ + A_γ_). Here, C_A_ and C_S_ are, respectively, the numbers of aromatic and saturate carbon atoms, whereas A_(002)_ and A_γ_ are, respectively, the areas of the graphene and γ bands.

**Figure 9:**
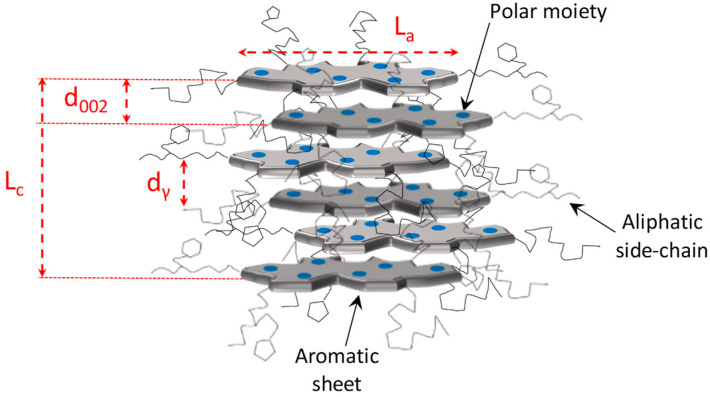
Lamellar configuration stabilized by intermolecular interactions to form stacked local discotic structures.

From the analysis of the XRD pattern, a lamellar configuration stabilized by π–π interactions was derived, as sketched in Figure 9, characterized by the interlayer distance (d_M_) and the distance between the saturated portions of the cluster (d_γ_). The thickness (L_c_) and diameter (L_a_) of the aromatic sheets can be estimated from the Scherrer equation $L_{j}=\frac{K_{j}\lambda }{\beta cos\theta_{j}}$, where *K_j_* is a shape factor (1.84 and 0.9 for L_a_ and L_c_, respectively), *β* is the line broadening, and *θ_j_* the position corresponding to the (10) and (002) bands, respectively, for the L_a_ and L_c_ lattice parameters [183,184,185].

Interestingly, both the γ and graphene (002) bands were also observed in liquid phase in the WAXS spectra [130].

Finally, SANS is a very functional, practical and potent technique for characterizing the suspension structure at the colloidal length scale. The neutron wavelength selected in SANS measurements can range from 1 to 30 Å, which is of a size similar to or smaller than that of the suspended particles. SANS analysis projects the intensity of the scattering as a function of the scattering angle (equal to the transfer of momentum), which carries the structure of the ASP aggregates (particles) and their interaction parameters. However, these parameters need to be extracted by proper data analysis, and special care should be taken in situations where models are used to analyze SANS data. In certain situations, an analysis scheme that is model-independent can only be used to analyze the data in a limited sense, such as surface to volume ratio, which facilitates the identification of the shapes of the particles. Scattering experiments have revealed that large ASP aggregates possess a very open (solvated), self-similar (fractal-like) internal structure [148,186,187].

The shape of ASP aggregates in organic solvents was found to be spherical [145,176,188] or cylindrical [155,156,158]. SANS measurements carried out on ASP dispersed in toluene solution revealed a continuous disaggregation process, reflected in a slow reduction in R_g_ at T > 300 K [177]. From the analysis of the scattered intensity in the small-q (Guinier) region acquired at T = 563 K, the authors calculated R_g_ = 35 Å. However, they were unable to establish whether that size matched the ASP basic units. Via SANS experiments, Tanaka et al. [189] demonstrated that ASP aggregates were prolate ellipsoid, with a high aspect ratio in some solvents at room T. However, upon increasing the temperature to 300 °C, the aggregates became more compactly packed and spherically shaped. The parallel semiaxes of the ellipsoids were about 150, 30, and 25 Å at 30, 150, and 300 °C, respectively. The size of the aggregate and its temperature dependency were different among different ASPs and solvents. For example, Maya ASP dispersed in decalin gave large fractal aggregates greater than 1000 Å at room temperature [189].

## 4. Structure of Bitumen Aggregates by Computer and Molecular Dynamics Simulations

Molecular dynamic (MD), molecular mechanical (MM), and quantum mechanical (QM) simulations provide a good opportunity to determine the energies at which the ASP self-association processes occur at the atomic level [190]. The simulation that serves to predict a result not obtained by conventional laboratory experiments aims to provide details on the precise structure and energy of association of ASP aggregates, although these can hardly cover the whole chemical complexity of the ASP system. In this regard, a well-established strategy is to perform computational techniques on model molecules that mimic ASPs [191]. As summarized in a recent review by Farooq et al. [192], a net dipole moment can arise from the structural arrangement of the atoms in the molecules, and/or the heteroatoms (nitrogen, sulfur, nickel, vanadium, etc.), which are often found in ASP. As soon as the net dipole moments of the stacks become non-zero, the process of nucleation is subdued, and aggregate growth is hindered and eventually halted. Indeed, Kusaka et al. [193], studying the effect of the dipole moment on the process of nucleation, concluded that the dipole moment needs to vanish for the process of nucleation to proceed. This could be why the phase separation phenomenon (commonly known as the process of aggregation) occurs, and as it vanishes on the colloidal length scale, it would come to resemble the process of micellization, although the energies involved are totally different.

**Figure 10:**
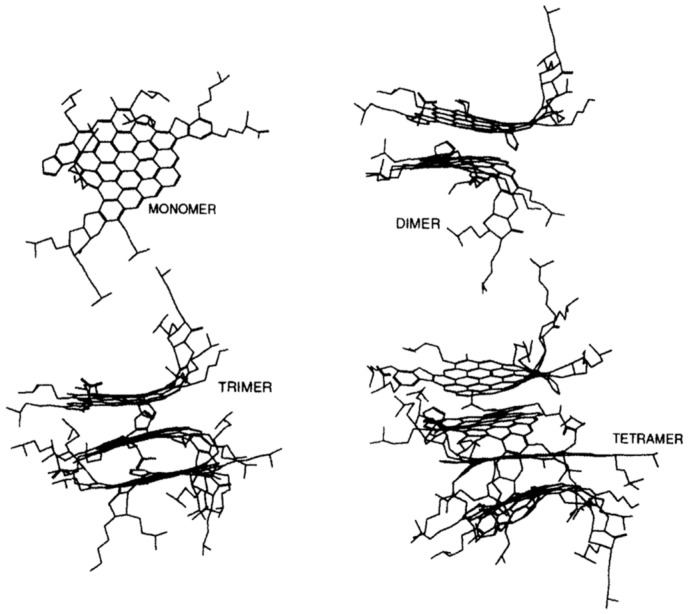
Simulated structures of asphaltene monomer and various aggregates consisting of an aromatic core surrounded by aliphatic chains [194].

One of the first pieces of evidence in favor of the colloidal model was provided by the pioneering studies of molecular dynamics calculations performed by Rogel [194], who showed that the interactions that occur between molecules with greater aromaticity and higher degrees of aromatic condensation could lead to the stabilization of the corresponding stacked aggregates (Figure 10).

Subsequent MD simulation studies under vacuum proved that the stacking of aromatic rings could occur for both large- and small-island (Yen–Mullins model) ASP model compounds [195,196,197]. The calculations made by Jian et al. [198] show that the presence of long sidechains had a detrimental effect on the stacking of aromatic cores, and at the same time promoted the aggregation phenomenon through hydrophobic association. The influence of temperature and solvent on the phenomenon of aggregation can also be simulated. For example, Zhang et al. [199] showed that the distance in the ASP dimer decreased with increasing temperature, while the simulation involving ASP molecules in various types of solvent revealed that the distance of aromatic core stacking in a bad solvent (heptane) was shorter than in a good solvent (toluene) [200]. Another noteworthy result obtained from molecular simulation is the calculation of ASP aggregate thermodynamic data. For example, the binding energies in ASP model compounds were found to be about 60 and 130 KJ/mol, respectively, via density functional theory (DFT) calculations [201] and the MM/MD computation of intermolecular potentials [202]. The discrepancy between the calculated binding energies is due to the different simulation methods employed and the different models of ASP structure. Sedghi et al. [203] demonstrated that as the number of aromatic rings increased, the association energy also increased substantially, and this was strongly affected by the heteroatoms, both those linked to aromatic cores and those attached to sidechains. da Costa et al. [204] subsequently carried out a more detailed DFT calculation, which evaluated the free energy of association of various kinds of aromatic hydrocarbons as model ASP compounds. The same authors, from DFT calculations of the interaction enthalpy (ΔH) and Gibbs free energy (ΔG) at 298 K, concluded that H-bonding was equally as important as that of aromatic ring stacking during the process of the formation of ASP aggregates [205]. The predictions of other properties, such as the molar volume, solubility parameter, cohesive energy, density, thermal expansion coefficient, enthalpy, and specific heat (at a constant pressure), of the model ASP structures have been also reported by using MM calculations and MD simulations [206,207,208]. The interactions between ASP and resins, water, inhibitors and other species have been investigated by the molecular simulation approach.

For example, Barcenas et al. [209,210,211] performed computer calculations by applying a particle agglomeration control (PAC) model with Monte Carlo simulation (PAC-MC). This model obtained consistent and trustworthy theoretical trends with respect to the concentration of ASP and the temperature, and allowed them to establish some variables that influence the efficiency of the inhibitor. The PAC-MC model was flexible enough to describe the average cluster size and the distribution of the ASP colloid aggregate cluster size, each having different origins. Besides this, the PAC-MC model generated a theoretical calculation, which demonstrated that an increment in the concentration of resins led to a reduction in the size of the ASP cluster.

For example, Alvarez-Ramirez et al. [201] used DFT to evaluate the interaction potential curves between ASP–resin, ASP–ASP, and resin–resin systems. Ortega-Rodríguez et al. [202] simulated the medium effect of a mixture of ASP and resins using QM/MM, and they were able to summarize the tendency of aggregation against the solvent polarity (heptane, toluene, and pyridine). One of the limitations of computational methods is that computer experiments, for the most part, still depend on experimental data, which might or might not be accurate. Thus, the conclusions arrived at from the computer simulations may still not accurately describe a system as complex as ASP. To overcome the difficulty of simulating complex crude oil mixtures directly at all atomic levels, which is well beyond current computational capabilities, several researchers have adopted the strategy of using coarse-grained (CG) descriptions, in which the groups of atoms are treated as a unit [212].

In this regard, it is worth mentioning dissipative particle dynamics (DPD), which is a CG particle model that facilitates, on a large time scale, the simulation of thousands of compounds [213]. The DPD approach allows for the study of ASP mesoscale behavior, as has been demonstrated by Zhang et al. [214], who proposed a detailed method that can be used to generate the average structure of the ASP molecule and also determine the DPD force parameters. The structure of the aggregate deduced from DPD was in concordance with MD/MM results and XRD data. The simulation of the aggregation and diffusion phenomena of ASP in heptane solvent was achieved by Wang et al. [215] using the DPD method. Dunn et al. [216] lately proposed a CG model to investigate the physical picture behind the Yen–Mullins model, and in more general terms, the influence that the molecular structure and solvent characteristics has upon the ASP aggregation phenomenon. They found that island-type ASPs are able to form nanoaggregates whose aromatic cores are stacked when subjected to conditions that favor a strong attraction between both aliphatic and aromatic sites. Contrarily, archipelago ASPs formed large aggregates under simulated conditions, which favored effective attraction between either aromatic or aliphatic sites. Although the DPD method is able to model a complex system, there are still two limitations to its application in ASP simulations. The first drawback is that an accurate composition input of ASP for the DPD model does not exist yet. Second, in order to further develop and provide accurate and reliable predictions of the molecular interactions and physical properties, accurate force parameters are needed. In a nutshell, simulations have one main difficulty: accurately imitating real systems. Regarding simulation experiments, in the set-up, the set of initial conditions has to be appropriate and precise, and to do this, in-depth knowledge of the system combined with a high level of technical know-how is fundamental.

## 5. Novel Challenges in Bitumen Research

The body of knowledge established so far on the structure of bitumen, represents a solid starting point able to potentially provide innovative and sustainable solutions in response to the needs of the road paving industry. In fact, the demand for bituminous products with reduced environmental impact, as required by the principles of circular economy, is increasingly insistent today. For example, the first challenge is to provide new technologies to regenerate aged bitumen from pavement waste materials.

As reclaimed asphalt pavement (RAP) is highly oxidized due to its long service life, reusing more than 20% of RAP by mixing it with fresh binder could increase the hardening of the bitumen, and consequently worsen the performance of the asphalt mixture itself. Recent studies have shown that the addition of suitable rejuvenating agents to RAP, yielding the correspondent rejuvenated binders, represents a promising strategy to make the performances of recycled bituminous mixtures comparable to those of the original material [217]. With this stratagem, it is possible to increase the fraction of RAP in the newly formulated mixtures to values higher than 20% [218].

Another consolidated research line focuses on the properties of polymer-modified bitumen (PMB) to improve the resistance of road pavements to thermal cracking and rutting, with the concomitant reduction in thermal susceptibility, aging, fatigue damage and stripping. In particular, inspired by environmental considerations, the current technology is aimed at reusing low quantities of recycled materials, such as crumb rubber from waste tires, in combination with one or more traditional road polymers to develop a hybrid recycled PMB [219,220]. The modification of bitumen by adding small amounts of styrene butadiene styrene (SBS), one of the most commonly used polymers in the PMB formulation, gives rise to numerous advantages, such as improved thermal susceptibility, increases in the softening point, and a significant reduction in the penetration value. Furthermore, it has been observed that SBS can mitigate the increase in bitumen stiffness as a consequence of the irreversible oxidative aging processes caused by long-term pavement service [221]. However, despite the proven benefits regarding the use of polymers in modified bitumen systems, several research studies have shown the difficulties regarding incompatibility with bitumen. Indeed, using a high percentage of crumb rubber in PMB can result in significantly lower workability, the separation of rubber–bitumen, and a decrease in the low-temperature performance of such modified bitumen.

Driven by the increasingly pressing search for sustainable materials from renewable resources to replace the by-products of petroleum refining, research efforts are also being directed towards the production of bio-oils or bio-binders via the transformation of waste biomass. Hence, several technologies, including pyrolysis, gasification, hydrothermal liquefaction and direct combustion, are currently being employed for converting biomass into bio-oil characterized by properties akin conventional bitumen [222]. Studies are trying to demonstrate the feasibility of hybrid bituminous binders, in which appropriate quantities of bio-oil and polymeric waste are added to virgin bitumen.

However, laboratory and field tests on the thermal susceptibility of these sustainable bituminous composites are still at an early stage, and we therefore have to await the outcome of further experimental investigations before these eco-friendly binders can make their appearance on the market of materials for road paving.

## 6. Conclusions

The search for a unique model that adequately describes the physicochemical properties of a complex system such as bitumen is still far from over. However, several points can be concluded on the state of the art of investigations on bitumen’s structure:

- A huge number of research reports have demonstrated that ASP aggregates exist on the colloidal length scale. One of the strengths of the colloidal model is the fact that ASP molecules are able to self-associate when in solution, in order to generate what we can call extended aggregates;
- Among the various efforts made to summarize the wide range of observations into a molecular picture, the colloidal model remains the most widely accepted and successful. This model assumes that polar molecules in crude oil residua interact to form molecular associations. These molecular associations are believed to be dispersed in a bulk solvent, which consists of saturated components, aromatic compounds, and less polar heteroatom-containing compounds;
- The size of the supramolecular associations, which are held together by forces varying in strength, is a function of the nature of the polar compounds, temperature, rate of shear, and the effectiveness with which the associations are solvated;
- Various properties of bitumen, such as its adhesion property or rheological behavior, are highly affected by the binder’s chemical composition and the macroscopic organization of ASP. However, not a lot is known about the relationship between the macroscopic organization of ASP and the rheological properties, resins and polymers that are added and incorporated into the bitumen matrix. The aging of bitumen is another important topic, and this is thought to be associated with the evolution of the ASP content and the macroscopic organization with time and exposure to oxidation.

Does the current research provide sufficient information on how these microstructures influence the rheological properties and aging resistance of bituminous binders, or how they affect pavement performance?

It is known that polar associations in bitumen are of great importance in influencing the binder properties, and it is important to introduce a model of bitumen structure in which molecular associations are central. Models that describe the structural arrangement of bitumen at the molecular level are required to explain its mechanical behavior in terms of its chemical composition.

In summary, the study of ASP aggregates in crude oils is significantly relevant to an array of phases, ranging from oil recovery via transport to refining. The in-depth study of the associations and interactions that exist or are created between ASP molecules/aggregates in an organic solvent sheds more light on the general understanding of the concept of ASP aggregation behavior in crude oils and in solvents. A concise understanding of ASP–ASP interactions, their aggregation, and their subsequent precipitation is inhibited by the lack of an accurate molecular identification of the individual ASP components. The experimental validation of the mechanisms of ASP assembly has, up to this point, proven elusive. These mechanisms of assembly and the nature of the subsequent supramolecular structures are still subjects of research that is currently ongoing.

## Figures and Tables

**Table 1:** Chronological evolution of different models that have appeared in the literature.

Modeling Approach	Main Features	Reference
colloidal	Bitumen is viewed as a colloidal dispersion of ASP micelles stabilized by resins	Nellensteyn [24] Pfeiffer et al. [25,26] Traxler et al. [27] Kawanaka et al. [28] Storm et al. [29] Lian et al. [30]
colloidal	ASPs are plate-like structures stacked together that form particles or crystallites	Yen et al. [31,32] Dickie et al. [33] Mullins [34]
solubility	ASPs are complexed by resins without forming micelles	Altegelt et al. [35] Koots et al. [36] Petersen [37] Christensen et al. [38]
solubility	Bitumen is a single-phase and homogenous fluid	Anderson et al. [39] Redelius [40]
colloidal	ASP micelles consist of an insoluble molecular core associated with surfactant-like resins in thermodynamic equilibrium	Lesueur [41] Rogel [42]
colloidal	Bitumen microstructure is formed by insoluble ASP aggregates stabilized by a fraction of more soluble ASP	Acevedo et al. [43,44,45]
colloidal	ASPs are stabilized by a series of nested shells with decreasing polarity	Wiehe et al. [46]
colloidal	Bitumen microstructure is constituted by ASP particles, micelles, and clusters of micelles held by molecular interactions	Yen et al. [47]
colloidal	Hierarchical microstructure of bitumen made by ASP molecules, nanoaggregates of ASP and cluster of nanoaggregates	Mullins et al. [48,49,50]
colloidal	Mechanism of micellization for ASP aggregates	Li et al. [51] Liu et al. [52]
solubility	Solution of ASP in oil is described in terms of thermodynamic liquid–liquid equilibrium	Wang et al. [53]
solubility	ASP association is interpreted according to a stepwise polymerization scheme	Agrawala et al. [54] Yarranton et al. [55]
colloidal	ASP nanoaggregates dispersed in solution generate structures with pores and host–guest complexes	Gray et al. [56]

ASP = asphaltene(s).

## List of Abbreviations

AFM	Atomic Force Microscopy
ASP	Asphaltene(s)
CAC	Critical Aggregate Concentration
CCC	Critical Cluster Concentration
CMC	Critical Micelle Concentration
CNAC	Critical Nanoaggregation Concentration
DPD	Dissipative Particle Dynamics
DPF	Dispersed Polar Fluid
FTIR	Fourier Transform Infrared spectroscopy
MW	Molecular Weight
NMR	Nuclear Magnetic Resonance
GPC	Gel Phase Chromatography
PAC	Particle Agglomeration Control
PMB	Polymer Modified Bitumen
R_g_	Radius of gyration
RAP	Reclaimed Asphalt Pavement
SANS	Small-Angle Neutron Scattering
SAXS	Small Angle X-ray Scattering
SARA	Saturate, Aromatic, Resin and Asphaltene
T_g_	Glass Transition Temperature
VPO	Vapor Pressure Osmometry
WAXS	Wide-Angle X-ray Scattering
XRD	X-ray Diffraction
